# Research hotspots and trends on neuromyelitis optica spectrum disorders: insights from bibliometric analysis

**DOI:** 10.3389/fimmu.2023.1135061

**Published:** 2023-07-13

**Authors:** Xin Chen, Jun Xiao, Luo-Qi Zhou, Wen-Xiang Yu, Man Chen, Yun-Hui Chu, Ke Shang, Gang Deng, Wen-Hui Song, Chuan Qin, Deng-Ji Pan, Dai-Shi Tian

**Affiliations:** Department of Neurology, Tongji Hospital, Tongji Medical College, Huazhong University of Science and Technology, Wuhan, China

**Keywords:** neuromyelitis optica spectrum disorders, bibliometric analysis, CiteSpace, VOSviewer, research hotspots

## Abstract

Neuromyelitis optica spectrum disorders (NMOSD) are demyelinating diseases of the central nervous system, have drawn the attention of many researchers due to the relapsing courses and cumulative disability. A first bibliometric analysis of NMOSD was conducted to identify the research hotspots and emerging trends. Articles relevant to NMOSD published in the core collection of Web of Science were retrieved and analyzed through visualized analysis using CiteSpace and VOSviewer, focusing on annual publication trends, countries, institutions, authors, journals, and keywords. The analysis showed that over the past 30 years, publications related to NMOSD had shown steady growth with slight fluctuations. The United States played an important part in this field, with the highest outputs and the greatest number of citations. Research hotspots of NMOSD had gradually shifted from the definition, biomarkers, and diagnostic criteria to diagnosis and treatment, particularly immunotherapy. This bibliometric analysis provides researchers with a theoretical basis for studying NMOSD and offers guidance for future research directions.

## Introduction

Neuromyelitis optica spectrum disorders (NMOSD) are immune-mediated demyelinating diseases primarily targeting the central nervous system, characterized by severe demyelination and axonal damage (1–3). The prevalence of NMOSD ranges from 0.7 to 10 per 100,000 in different populations worldwide, with the incidence in women significantly higher than in men (4, 5). Relapsing courses and cumulative disability reduce the quality of life in patients and impose great burdens on families and society, which have drawn the attention of many researchers. Bibliometric analyses have been performed in certain professional fields, such as cardiovascular disease (6, 7), infectious diseases (8, 9), and oncology (10–12). In contrast, bibliometric analysis of NMOSD is rare. This bibliometric analysis aims to elucidate the research hotspots and new trends of publications over the past 30 years.

## Methods

### Data acquisition

The eligible studies published until 15 October 2022 were downloaded from the core collection of Web of Science. The main search terms were as follows: TS=Neuromyelitis Optica Spectrum disorder* OR Neuromyelitis Optica OR NMOSD OR NMO. The search was restricted to articles and reviews in English. The time span of this analysis was from 1990 to 2022. Two researchers retrieved and screened the raw data independently and then cleared divergences. The detailed screening process is shown in **Supplementary Figure 1.**

### Data analysis and visualization

Data analysis and visualization were performed utilizing CiteSpace (version 6.1.R3) and VOSviewer (version 1.6.18). Microsoft Excel summarized the annual number of publications (Np) and plotted the bar chart. CiteSpace and VOSviewer were used to visualize the co-occurrence of authors, countries, journals, institutions, keywords, clusters and burst of keywords, and co-cited authors, co-cited journals, and co-cited references.

Visualization networks were composed of nodes and lines generally. The node size signified the number of publications, and the number of lines among the nodes represented cooperation intensity. In light of burst keywords, a red line segment was used to represent the period when a keyword was calculated with the strongest citation burst (13). The impact factors (IF) of journals were acquired from the latest Journal Citation Reports (JCR) 2021 in Web of Science.

## Results

### Annual growth trend of publication outputs

**Figure 1:**
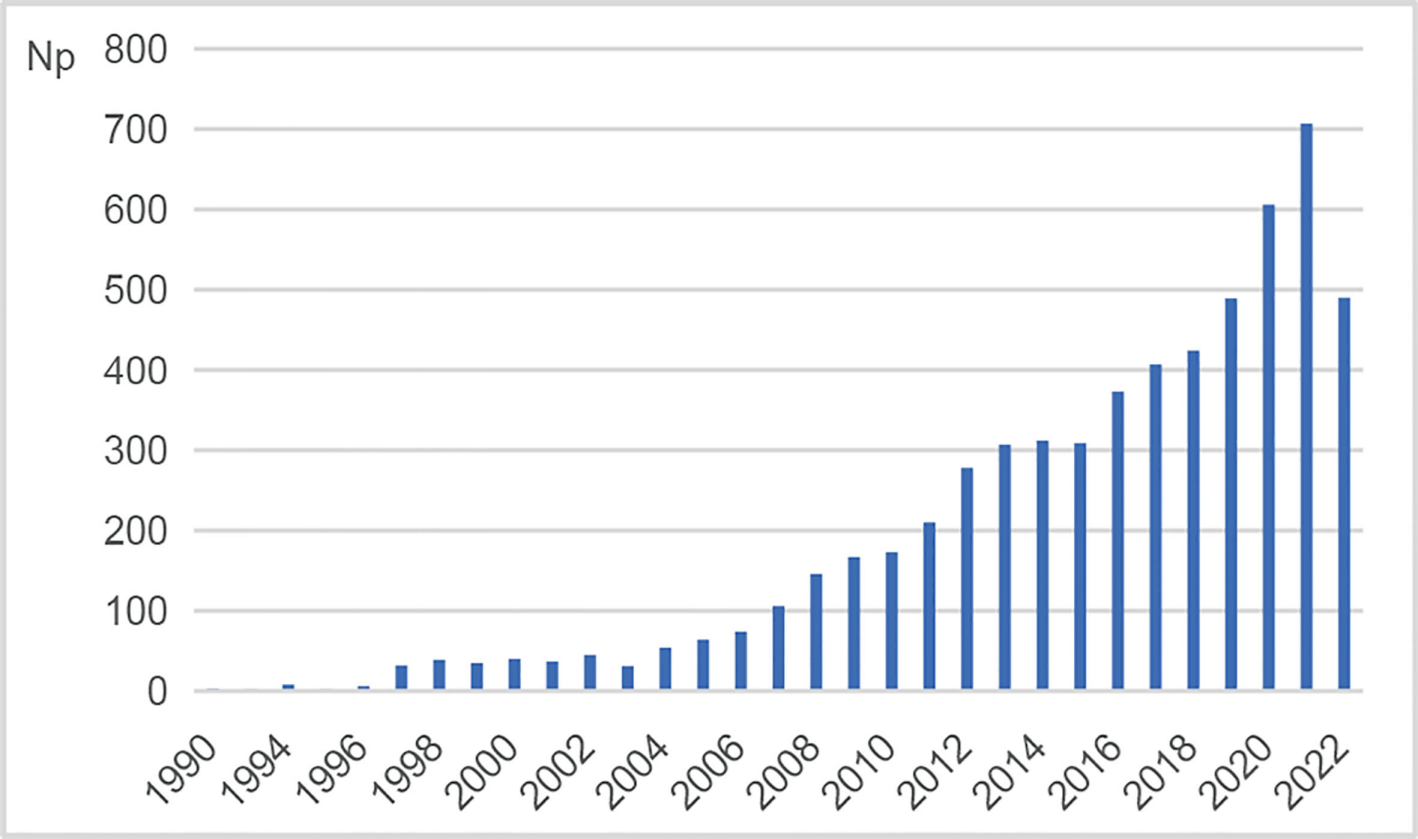
Annual number of publications (Np) on NMOSD research. NMOSD, neuromyelitis optica spectrum disorder.

The retrieval identified a total number of 5,974 publications restricted to articles and reviews in this field from 1990 to 2022. The yearly growth of publications reflected the growing trend of research. **Figure 1** shows the annual number of publications (Np) on NMOSD. In general, there was an upward trend, despite slight fluctuations. It showed that Np was low from 1990 to 2006, which might result from the lack of understanding of the disease. With the discovery of pathogenic antibodies, the update of diagnostic criteria, and the development of new drugs, an increasing number of scholars concentrated on exploring the etiology, pathogenesis, clinical manifestation, diagnosis, treatment, and prevention of NMOSD. As a result, Np showed a tremendous increase from 2007 to 2021, especially more than 700 in 2021, indicating a rapid development in this field.

### Countries

**Figure 2:**
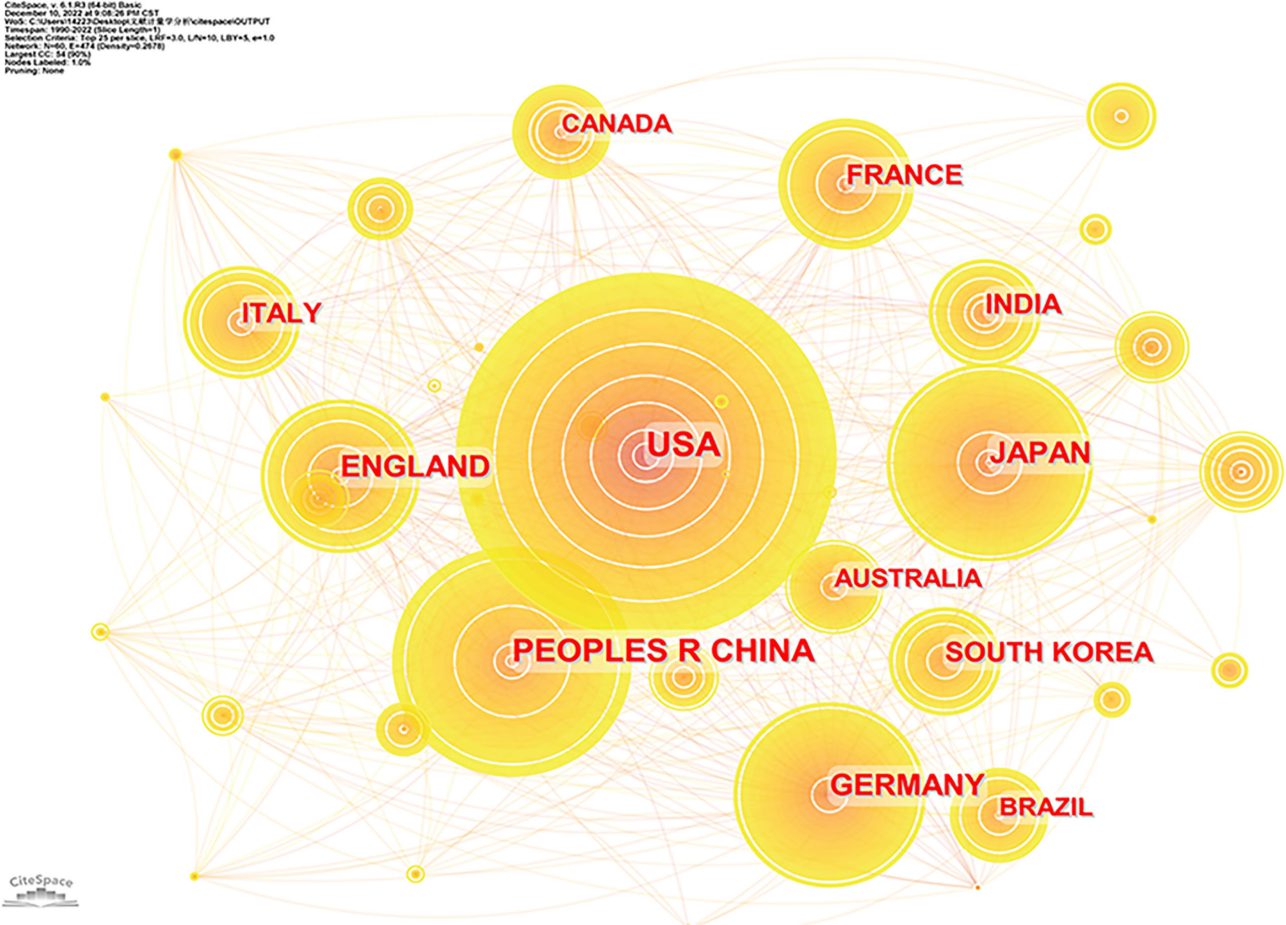
Co-occurrence of countries involved in the study of NMOSD. NMOSD, neuromyelitis optica spectrum disorder.

There were 122 countries involved in publishing articles on NMOSD in the past 30 years. **Figure 2** shows the co-occurrence of countries, and **Table 1** lists the top 10 countries that delivered the most articles with their number of citations (Nc) and total link strength (TLS). The United States published most of the articles (1,610), followed by China (1,019) and Japan (621). TLS represented cooperation intensity with each other. In terms of TLS, the United States had the highest TLS (1,468), implying the strongest cooperation with others, followed by Germany (1,179) and the UK (1,127).

**Table 1:** Top 10 countries with the most publications about NMOSD.

Rank	Country	Np	Nc	TLS
1	USA	1,610	88,946	1,468
2	China	1,019	12,389	341
3	Japan	633	34,763	535
4	Germany	601	39,178	1,179
5	England	500	44,298	1,127
6	France	325	29,206	681
7	Italy	311	23,939	625
8	South Korea	284	7,311	345
9	India	267	4,330	219
10	Brazil	240	7,508	385

NMOSD, neuromyelitis optica spectrum disorder; Np, number of publications; Nc, number of citations; TLS, total link strength.

### Institutions

**Supplementary Figure 2** shows the co-occurrence of institutions, and **Table 2** lists the top 10 institutions in light of the outputs of NMOSD. These institutions were dispersed in the United States (3), Japan (3), China (2), Germany (1), and Austria (1). Mayo Clinic (247) published the largest number of articles on NMOSD; Tohoku University (202) and the University of California—San Francisco (153) followed closely. The top three institutions regarding TLS were Charité – Universitätsmedizin Berlin (763), Tohoku University (654), and Mayo Clinic (610). As the data showed, there was considerably close cooperation between institutions.

**Table 2:** Top 10 institutions with the most publications about NMOSD.

Rank	Institution	Country	Np	Nc	TLS
1	Mayo Clinic	USA	247	34,236	610
2	Tohoku University	Japan	202	20,196	654
3	University of California—San Francisco	USA	153	18,320	397
4	Sun Yat-Sen University	China	152	2,202	152
5	University of Oxford	England	128	9,354	446
6	Capital Medical University	China	122	1,482	153
7	Charité – Universitätsmedizin Berlin	Germany	110	4,317	763
8	Johns Hopkins University	USA	94	5,809	320
9	Medical University of Vienna	Austria	93	7,229	356
10	National Cancer Center	Japan	88	3,665	336

NMOSD, neuromyelitis optica spectrum disorder; Np, number of publications; Nc, number of citations; TLS, total link strength.

### Authors and co-cited authors

In total, 21,328 authors contributed to the publications related to NMOSD. **Supplementary Figure 3** shows the co-occurrence network of authors, and **Table 3** lists the top 10 authors who produced the most publications of NMOSD. Fujihara, Kazuo (115), ranking first, had the highest Np with 13,886 citations, followed by Paul, Friedemann (112), and Takahashi, Toshiyuki (108). However, in terms of TLS, Fujihara, Kazuo (770), Paul, Friedemann (756), and Nakashima, Ichiro (576) ranked the top 3 among the top 10, implying that these authors had closer cooperation with others. Notably, the top 10 authors were mainly from the United States and Japan.

**Table 3:** Top 10 authors with the most publications about NMOSD.

Rank	Author	Country	Np	Nc	TLS
1	Fujihara, Kazuo	Japan	115	13,886	770
2	Paul, Friedemann	Germany	112	5,602	756
3	Takahashi, Toshiyuki	Japan	108	3,948	499
4	Qiu, Wei	China	92	1,327	536
5	Nakashima, Ichiro	Japan	85	3,761	576
6	Kim, Ho Jin	South Korea	84	2,975	445
7	Levy, Michael	USA	80	4,954	311
8	Pittock, Sean J.	USA	79	5,729	500
9	Palace, Jacqueline	England	74	4,934	532
10	Weinshenker, Brian G.	USA	70	9,535	419

NMOSD, neuromyelitis optica spectrum disorder; Np, number of publications; Nc, number of citations; TLS, total link strength.

Cited authors who appeared concurrently in references were named co-cited authors. Among the 71,271 co-cited authors, 263 were co-cited more than 100 times, and six were co-cited over 1,000 times. The co-occurrence of co-cited authors is visualized in **Supplementary Figure 4**, and the top 10 co-cited authors are displayed in **Supplementary Table 1**. Wingerchuk, Dean M. (6,333) was co-cited at most, followed by Jarius, Sven (3,737), and Lennon, Vanda A. (2,808).

### Journals and co-cited journals

In this research, 5,974 articles regarding NMOSD were delivered in 1,188 academic journals, of which the highest IF surpassed 50 (*Lancet Neurology*). *Multiple Sclerosis and Related Disorders* ranked first in terms of articles (374), followed by *Multiple Sclerosis Journal* (295) and *Journal of Neuroimmunology* (205). Six were in the Q1 JCR partition, and three had an IF over 10 among the top 10 journals (**Supplementary Table 2**).

The co-citation analysis of journals showed that *Neurology* (22,933 citations) was cited the most, followed by *Multiple Sclerosis Journal* (10,386 citations) and *Annals of Neurology* (8,817 citations) (**Table 4** and **Supplementary Figure 5**). Of the total 13,258 journals, 33 had been co-cited more than 1,000 times, and six journals exceeded 5,000 co-citations. Of the top 10 co-cited journals, *Lancet Neurology* had the highest IF (59,935), followed by *Brain* (15.255). All of the top 10 co-cited journals except one (*Journal of the Neurological Sciences*) were divided in the Q1 JCR partition.

**Table 4:** Top 10 journals with the most published research on NMOSD.

Rank	Journal	Np	Nc	IF (2021)	JCR
1	*Multiple Sclerosis and Related Disorders*	374	3217	4.808	Q2
2	*Multiple Sclerosis Journal*	295	10,643	5.855	Q1
3	*Journal of Neuroimmunology*	205	3,018	3.221	Q3
4	*Journal of the Neurological Sciences*	154	3,690	4.553	Q2
5	*Neurology*	129	18,308	11.8	Q2
6	*Frontiers in Neurology*	126	1,025	4.086	Q1
7	*Journal of Neurology*	112	3,845	6.682	Q1
8	*Neurology-Neuroimmunology & Neuroinflammation*	85	2,333	11.36	Q1
9	*Frontiers in Immunology*	83	1,248	8.786	Q1
10	*Journal of Neurology Neurosurgery and Psychiatry*	73	3,559	13.661	Q1

NMOSD, neuromyelitis optica spectrum disorder; Np, number of publications; Nc, number of citations; IF, impact factor; JCR, Journal Citation Reports.

A dual-map overlay of journals on NMOSD was constructed by CiteSpace to explore the subject distributions of scholarly journals (14). There were two main regions on the overlay, with the left areas representing distributions of citing journals and the right representing cited journals. As we could see from **Supplementary Figure 6**, the citing journals mainly belonged to the areas labeled Medicine, Medical, Clinical and Molecular, Biology, Immunology, and Neurology, Sports, Ophthalmology, whereas the cited journals primarily focused on the fields labeled Molecular, Biology, Genetics, and Health, Nursing, Medicine.

### Co-occurrence, clusters, and burst of keywords

**Figure 3:**
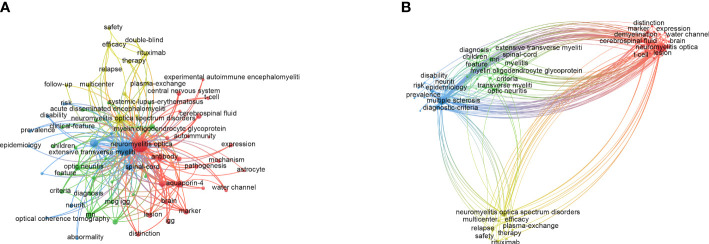
The maps of keywords appearing more than 100 times in the study of NMOSD. **(A)** Network diagram of keywords. **(B)** Cluster analysis of co-occurring keywords. NMOSD, neuromyelitis optica spectrum disorder.

Keywords generalized the main research contents of the articles. Therefore, the keyword co-occurrence network could present research topics intuitively in a certain field (15). In this research, the co-occurrence map of keywords was derived from VOSviewer. With restrictions on keywords that occurred more than 100 times, a total of 68 keywords were selected (**Figure 3A**). “Neuromyelitis optica” (3,077), “multiple sclerosis” (2,637), “anti-aquaporin 4 antibody” (1,535), “neuromyelitis optica spectrum disorder” (1,007), and “diagnostic criteria” (808) were the top 5 keywords used frequently. Along with the keyword co-occurrence, a density visualization map was also constructed, where keywords were colored according to the occurrence times (**Supplementary Figure 7**). The larger the number of keywords counted, the closer the color of nodes was to red. On the contrary, the smaller the number of keywords counted, the closer the color of nodes was to blue.

Keyword cluster analysis aimed to display the distribution of core contents, which classified keywords based on the similarity degree (16). As shown in **Figure 3B**, 68 keywords that occurred more than 100 times were grouped into four clusters: cluster 1 (red nodes, 24 items) mainly focused on mechanism and biomarkers such as anti-aquaporin 4 antibody (AQP4-IgG) and cytokines; cluster 2 (green nodes, 20 items) was mainly related to clinical features, other antibodies such as myelin oligodendrocyte glycoprotein antibody, and other demyelination diseases such as acute disseminated encephalomyelitis; cluster 3 (blue nodes, 12 items) concentrated on diagnostic criteria and the manifestation of auxiliary examination such as magnetic resonance imaging and optical coherence tomography; cluster 4 (yellow nodes, 12 items) was mainly related to the treatment of NMOSD, especially in the field of immunotherapy such as immunosuppressants and monoclonal antibodies.

**Figure 4:**
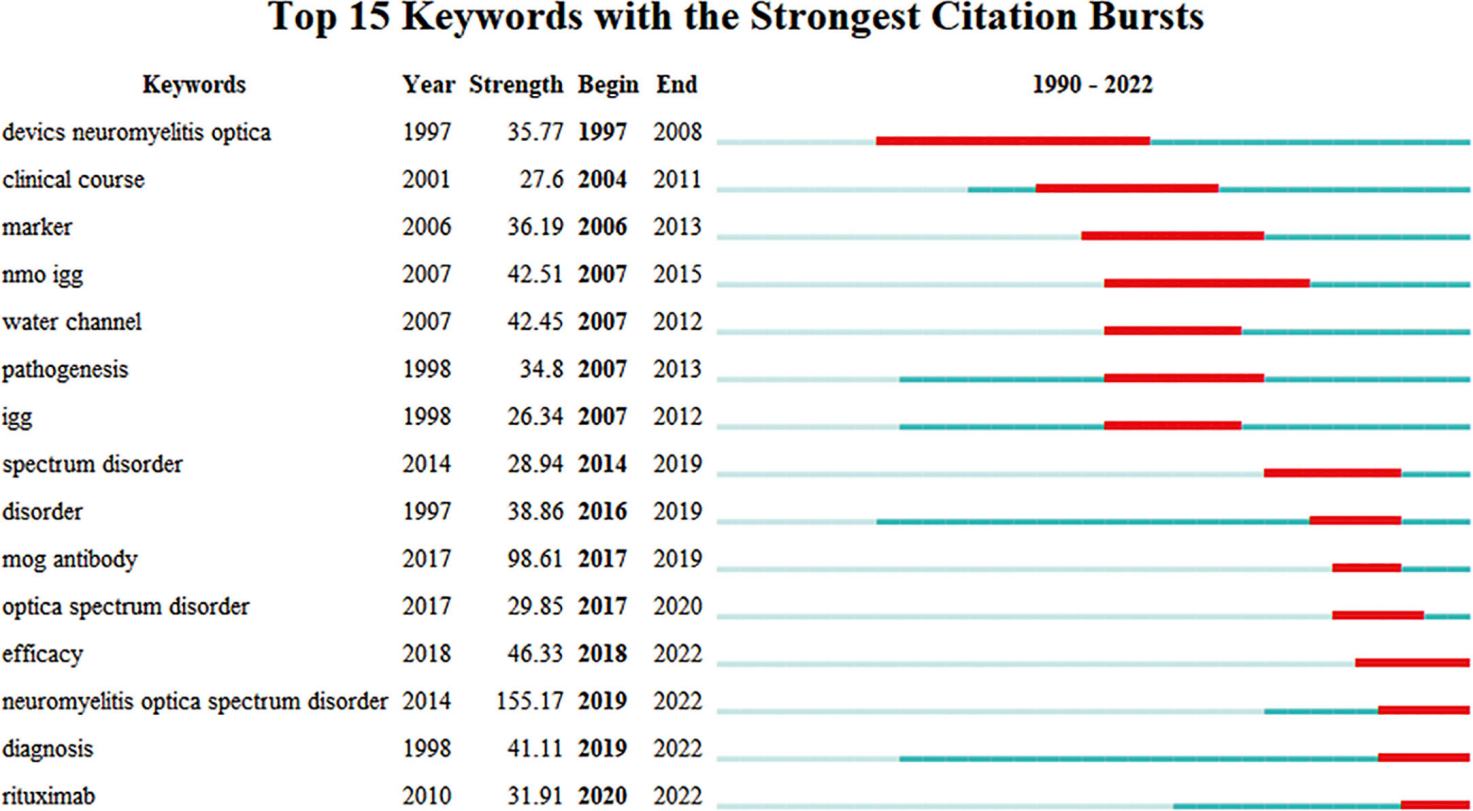
The top 10 keywords with the strongest citation bursts from 1997 to 2022 (generated by CiteSpace).

Burst keywords were considered another crucial indicator of research frontiers (13). Burst keywords referred to items that increased rapidly in frequency in a short time, the analysis of which reflected the evolution trend of research hotspots (16). The top 15 burst keywords are displayed after a thorough analysis in **Figure 4**. Burst keywords of NMOSD mainly focused on the definition and biomarkers in the early years. In recent years, the diagnosis and treatment of NMOSD have become new research hotspots in this field, and the burst is still ongoing.

### Co-cited references

Co-cited references referred to two articles that were cited simultaneously, and co-citation was confirmed by calculating the frequency of co-cited references (16). **Supplementary Figure 8** shows the co-occurrence of co-cited references, and **Supplementary Table 3** displays the top 10 co-cited articles whose co-citation appeared 423 times at least. The most co-cited reference on NMOSD was “International consensus diagnostic criteria for neuromyelitis optica spectrum disorders”, authored by Wingerchuk, Dean M. and published in *Neurology*, followed by an article entitled “A serum autoantibody marker of neuromyelitis optica: distinction from multiple sclerosis”. In general, two of the top 10 co-cited publications were documents about the diagnostic criteria, and three were related to AQP4-IgG, a unique biomarker of NMOSD.

## Discussion

Until now, this is the first comprehensive bibliometric analysis of NMOSD. In this analysis, we chose VOSviewer and CiteSpace to analyze 5,974 documents on NMOSD research filtered from the core collection of Web of Science to discover research hotspots and trends in this field.

As shown in **Figure 1**, the annual number of publications ranged from 1 to 707 during the period from 1990 to 2021. The number of publications showed an overall upward trend in the past 30 years despite slight fluctuations, implying that this field had gradually entered into a mature phase of development (7).

The top 3 high-yield countries listed were the United States, China, and Japan. The United States, with the strongest TLS, had the highest productivity. Three of the top 10 institutions with the most published research on NMOSD were situated in the United States. In addition, three of the top 10 authors who published the most articles on NMOSD also came from the United States. Thus, it turned out that the United States occupied a leading position in the research area, and there were still differences among nations and regions in the development of this area. Compared to the United States, China had lower Np, Nc, and TLS. It is noteworthy that China started to step into this field in 2005 but ranked second in gross outputs at present, which represented that China had made great contributions to NMOSD research in the past 15 years, which may be triggered by the higher prevalence.

We also explored whether there was a possible relationship between the prevalence of NMOSD and the number of publications. According to previous population-based studies about global epidemiology, the prevalence of NMOSD under 2015 diagnostic criteria ranged from 0.7 to 10/per 100,000 persons in different populations worldwide (5). Africans had the highest prevalence, whereas Whites had the lowest (17). There was also an increased prevalence of NMOSD in Asians and Africans (5). In terms of countries, the top three productive countries were the United States, China, and Japan. The prevalence of NMOSD in East Asia, such as China and Japan, is higher than that in the United States, so it seems that there is no linear correlation between prevalence and the number of publications. The high prevalence of a certain disease in a particular region is bound to attract the attention of researchers and lead to more efforts being devoted to its study. On account of the higher prevalence but lower productivity, countries in Africa and Asia are supposed to devote more energy to NMOSD research and strengthen their international cooperation.

As an indispensable approach to research dissemination, the quality and prestige of journals play a crucial part in transmitting research results (7, 18). In this research, *Multiple Sclerosis and Related Disorders*, *Multiple Sclerosis Journal*, and *Journal of Neuroimmunology* published the most articles over 200. These journals occupied an important position in this field. We manually collected the publication dates of those documents and identified the duration as 2003–2022. During the first decade when the disease emerged, there were no significant publications in the aforementioned journals, possibly due to a lack of awareness regarding these disorders. In terms of co-cited journals, *Neurology*, *Multiple Sclerosis Journal*, and *Annals of Neurology* were cited more than 8800 times. These journals that were all in the Q1 JCR partition might declare consensus and unique insights better.

According to the authors’ analysis, Kazuo, Fujihara, from Japan made the greatest contributions with 115 publications. He was a member of Fukushima Medical University, and his team focused on treatment for NMOSD. His latest research reviewed therapeutic approaches for symptomatic treatment and function restoration in NMOSD, such as remyelinating agents and mesenchymal stem cell transplantation (19), which provided new alternatives for NMOSD restoration treatment. In the co-cited author analysis, the most productive author was Wingerchuk, Dean M., from the United States. He made great contributions to the development of NMOSD, and his articles had been delivered in many authoritative journals such as *Lancet* and *Neurology*. His latest network meta-analysis suggested that the complement component C5 inhibitor eculizumab may be an effective approach to decreasing NMOSD relapses (20). Furthermore, the most co-cited reference produced by Wingerchuk, Dean M., was entitled “International consensus diagnostic criteria for neuromyelitis optica spectrum disorders” (2). This record updated the diagnostic criteria detailedly and laid a theoretical foundation for subsequent studies. The second most co-cited article was original research on a highly specific IgG autoantibody (NMO-IgG) (21). This study evaluated the ability of NMO-IgG to differentiate NMOSD from multiple sclerosis (MS). The detection of NMO-IgG allowed for early diagnosis, which in turn facilitated the timely implementation of appropriate immunosuppressive treatment.

The co-occurrence analysis of keywords played a significant role in data visualization. The findings revealed the research focus and evolution of keywords related to NMOSD (13). The results of keyword cluster analysis revealed four significant research orientations in the field of NMOSD: pathogenesis, biomarkers, diagnosis, and treatment. The variation trend of burst keywords indicated that the main research focus had shifted from biomarkers, which were the primary area of obsession in the early stages, to treatment in recent years. In 1894, French neurologists Eugène Devic and Fernand Gault first described published cases of optic neuritis with myelitis and named the disease neuromyelitis optica (NMO) or Devic’s disease (22). Since then, NMO gained the attention of scholars. However, distinguishing NMO from MS was difficult for a long time due to the similarity in clinical manifestations (23, 24). In 2004, the discovery of pathogenic AQP4-IgG, a highly sensitive and specific serum biomarker of NMO, made it possible to distinguish NMO from classic MS (24, 25). In 2007, Wingerchuk D. M. et al. first put forward the concept of NMOSD (26). With the incremental understanding of the disease, the International Panel for NMO Diagnosis (IPND) revised diagnostic criteria and identified the medical term NMOSD in 2015 (2). AQP4-IgG was considered one of the most important criteria for disease diagnosis. Until now, we still use the term NMOSD and the updated diagnostic criteria. In the past decade, the discovery of other antibodies, such as myelin oligodendrocyte glycoprotein antibody (MOG IgG) (27) and glial fibrillary acidic protein antibody (GFAP IgG) (28), has broadened our perception of demyelinating diseases of the central nervous system. Recently, due to the relapse courses, cumulative disability, and poor survival quality of NMOSD patients, scholars have devoted themselves to elucidating the possible pathogenesis (29) and have raised widespread concern regarding treatment, particularly immunotherapy.

It is of great importance to initiate appropriate treatment early in NMOSD management. Acute attacks or relapses are usually treated with intravenous glucocorticoids, which should be started early and aggressively to hasten recovery (1). Plasma exchange or intravenous immunoglobulin can be used as adjunctive or alternative therapies to relieve symptoms for patients who are not sensitive to glucocorticoids (30). The pivotal goals during the remission period are to minimize disability and prevent relapses. Immunotherapy plays a crucial part in these purposes. Currently, there is no drug that can completely prevent a recurrence, but a variety of immunosuppressants and monoclonal antibodies can significantly reduce the relapse frequency of NMOSD. Recently researchers have conducted numerous clinical trials of immunotherapy to verify the efficacy and adverse of new drugs (31–36). As a result, the most commonly used off-label drugs are azathioprine, mycophenolate mofetil, and rituximab. Rituximab has shown the most robust efficacy. With the discovery of AQP4-IgG and astrocytopathy, the era of targeted therapy is burgeoning, which has led to the development of a wealth of monoclonal antibodies targeting B cells, the complement system, and the IL-6 receptor (37). Four randomized placebo-controlled trials, namely, the PREVENT trial (38), the N-MOmentum trial (34), the SAkuraSky study, and the SAkuraStar study (32, 39), have demonstrated the efficacy of three new agents (eculizumab, satralizumab, and inebilizumab) for NMOSD patients. These three monoclonal antibodies have been approved by Food and Drug Administration for NMOSD treatment currently (40). Furthermore, several new agents such as aquaporumab, bortezomib, bevacizumab, ublituximab, and sivelestat are under development (41, 42), and they are expected to be approved in the future. Although all of the agents mentioned are effective in principle, it is difficult to widely recommend them due to factors such as their high cost and adverse effects. The most common adverse effects are infections, cytopenias, and infusion-related reactions (43). Therefore, it is important to continue researching more precise immunotherapy targeting antibody-producing cells in the near future.

More importantly, hematopoietic stem cell transplantation (HSCT) and chimeric antigen receptor T-cell therapy (CAR-T) (36, 44), novel approaches to reconstructing immune tolerance, are arousing great concern among a large number of scholars. Richard et al. reported that patients with relapsed or refractory NMOSD had significant improvements in the Expanded Disability Status Scale (EDSS), neurologic rating scale (NRS), and quality of life after HSCT (45). Recently, our teams, for the first time, have evaluated the safety and efficacy of CAR-T therapy in relapsed or refractory patients with AQP4-IgG-seropositive NMOSD in a phase 1 clinical trial (46). However, treatment evidence is insufficient, and more clinical trials are indispensable to verify the efficacy and safety of these new strategies. Therapeutic exploration relevant to these new methods will emerge as research hotspots in the future. These suggest that treatment has gained a large amount of attention recently and will remain a research priority in the future. However, the complicated pathogenesis but limited treatment lead to partial effectiveness (47). Yang et al. found a potential association between molecular mechanisms underlying NMOSD and proteins encoded by some novel genes (48) and more promising therapies such as gene therapies need to be exploited. However, the immunotherapy mentioned is mainly approved for AQP4-IgG-seropositive NMOSD, and options for seronegative patients are still limited because of obscure pathogenesis. It is not only a conundrum but also a challenge we are supposed to conquer in the future (42).

Furthermore, cumulative disability resulting from relapse requires the prediction of relapse risk, which is essential in guiding the early implementation of rational individualized treatment. Previous studies have reported various relapse predictors, such as female, younger age at onset, European or African descent, coexisting medical conditions, longer lesion length, and serum biomarkers such as GFAP and neurofilament light chain levels (42, 49–51). Wang et al. identified some relapse risk factors and developed an outcome prediction model comprised of gender, AQP4-IgG titer, previous attack under the same therapy, baseline EDSS score, and maintenance therapy to estimate the 1-year and 2-year relapse-free probabilities. The feasibility of this model was confirmed by multicenter cohort external validation (52). However, different predictors have presented inconsistent effects on relapse in various publications. In the future, it is necessary to conduct numerous clinical studies to explore and identify more predictors and establish a convenient and feasible relapse prediction model based on easily obtained clinical indicators. This will enable the assessment of relapse risk earlier, facilitate the generation of an individualized treatment strategy, and help avoid unfavorable outcomes.

## Limitations

There were several limitations to this analysis. First, raw data were only collected from the core collection database of the Web of Science, resulting in the loss of some relevant records. Therefore, in the future, we should retrieve data from additional databases to obtain a more comprehensive picture. Second, we discarded several non-English articles, which might have resulted in missing some critical studies on NMOSD. Finally, as with other bibliometric analyses (7, 13, 16), there may be biases in the analysis results due to the combined utilization of CiteSpace and VOSviewer.

## Conclusion

To summarize, this bibliometric analysis provided a comprehensive overview of NMOSD research for the first time. The study included a visualized analysis of NMOSD research by pooling output over the past three decades, covering annual trends of publications, global cooperation, research hotspots, and emerging trends. However, domestic research is still in the early stage of development. Therefore, more attention should be paid to the diagnosis and treatment of NMOSD, and conducting more clinical trials should be considered as the primary research direction and frontier in the future.

## Author contributions

D-ST, D-JP, and CQ conducted the conceptualization. D-ST, CQ, and JX identified the methodology. XC, W-XY, and W-HS were responsible for the screening of documents and visualization of the analysis. XC and L-QZ finished the images together. XC wrote the original manuscript. D-ST and CQ revised and retouched the manuscript. All authors contributed to the article and approved the submitted version.
