# Supplementary material for: Research hotspots and trends on neuromyelitis optica spectrum disorders: insights from bibliometric analysis
Source: Front Immunol. 2023 Jul 13;14:1135061. doi: 10.3389/fimmu.2023.1135061 (PMC10373306; doi:10.3389/fimmu.2023.1135061)
Supplement: Supplementary file 1 [file DataSheet_1.docx]

***Supplementary Materials***

**Research spots and Trends on Neuromyelitis Optica Spectrum disorders: Insights from Bibliometric Analysis**

Xin Chen^†^, Jun Xiao^†^, Luo-Qi Zhou, Wen-Xiang Yu, Man Chen, Yun-Hui Chu, Ke Shang, Gang Deng, Wen-Hui Song, Chuan Qin*, Deng-Ji Pan*, Dai-Shi Tian*

Department of Neurology, Tongji Hospital, Tongji Medical College, Huazhong University of Science and Technology, Wuhan 430030, China

^†^These authors contributed equally to this work.

* Correspondence to:

Dai-Shi Tian MD, PhD E-mail: [tiands@tjh.tjmu.edu.cn](mailto:tiands@tjh.tjmu.edu.cn)

OR

Deng-Ji Pan MD, PhD E-mail: [djpan@tjh.tjmu.edu.cn](mailto:djpan@tjh.tjmu.edu.cn)

OR

Chuan Qin MD, PhD E-mail: [chuanqin@tjh.tjmu.edu.cn](mailto:chuanqin@tjh.tjmu.edu.cn)

Department of Neurology, Tongji Hospital, Tongji Medical College, Huazhong University of Science and Technology, Wuhan 430030, P.R. China

Phone: +86-27-83663337

**1.Supplementary Figure**

Supplementary Figure 1. Detailed process for literature screening.

Supplementary Figure 2. Co-occurrence of institutions involved in the study of NMOSD.

Supplementary Figure 3. Co-occurrence of authors involved in the study of NMOSD.

Supplementary Figure 4. Co-occurrence of co-cited authors involved in the study of NMOSD.

Supplementary Figure 5. Co-occurrence of co-cited journals involved in the study of NMOSD.

Supplementary Figure 6. A dual-map overlay of journals on NMOSD.

Supplementary Figure 7. Density visualization map of keywords appearing more than 40 times in the study of NMOSD.

Supplementary Figure 8. Co-occurrence of co-cited references involved in the study of NMOSD.

**2.Supplementary Table**

Supplementary Table 1. Top 10 co-cited authors of publications about NMOSD.

Supplementary Table 2. Top 10 co-cited journals of publications about NMOSD.

Supplementary Table 3. Top 10 co-cited references of publications about NMOSD.


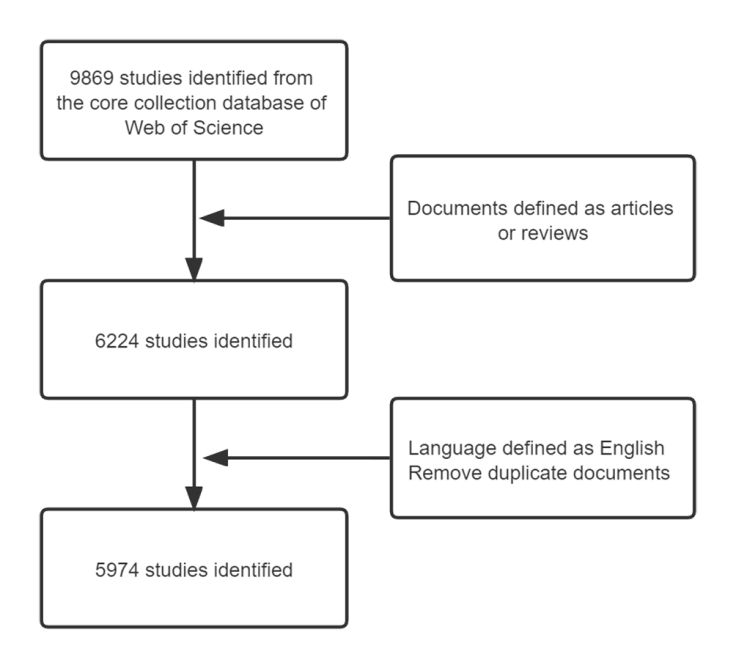


Supplementary Figure 1. Detailed process for literature screening.


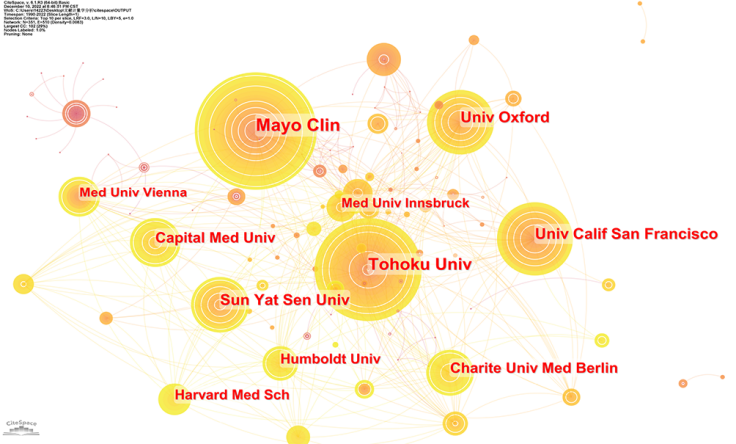


Supplementary Figure 2. Co-occurrence of institutions involved in the study of NMOSD.


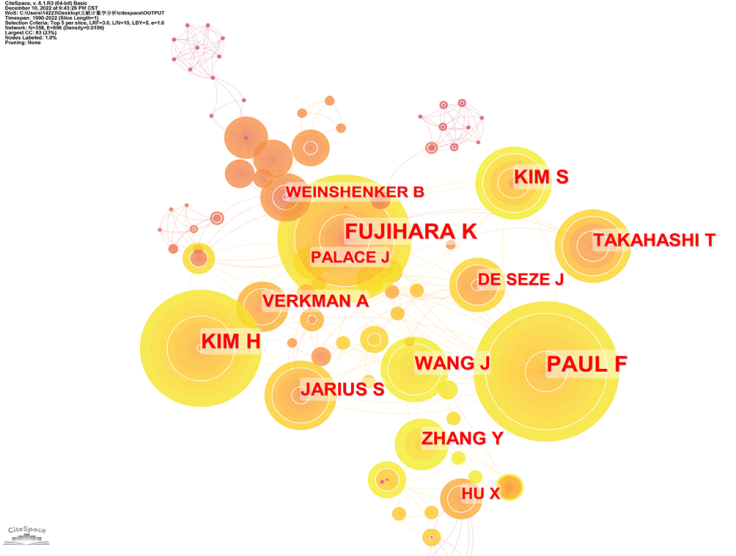


Supplementary Figure 3. Co-occurrence of authors involved in the study of NMOSD.


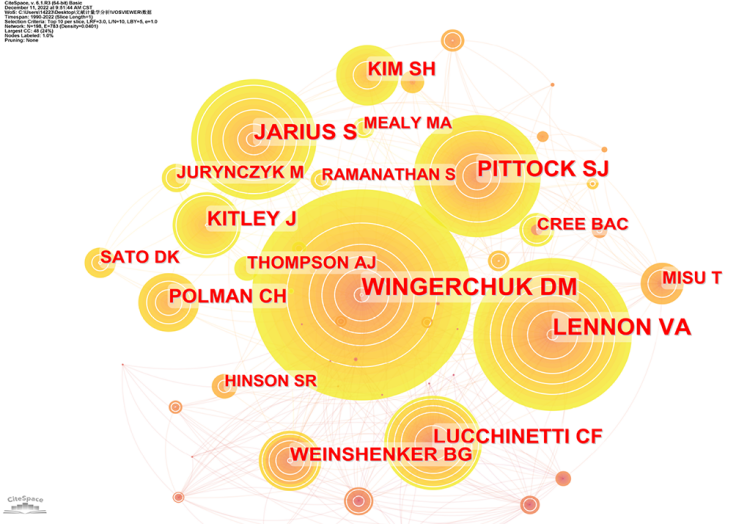


Supplementary Figure 4. Co-occurrence of co-cited authors involved in the study of NMOSD.


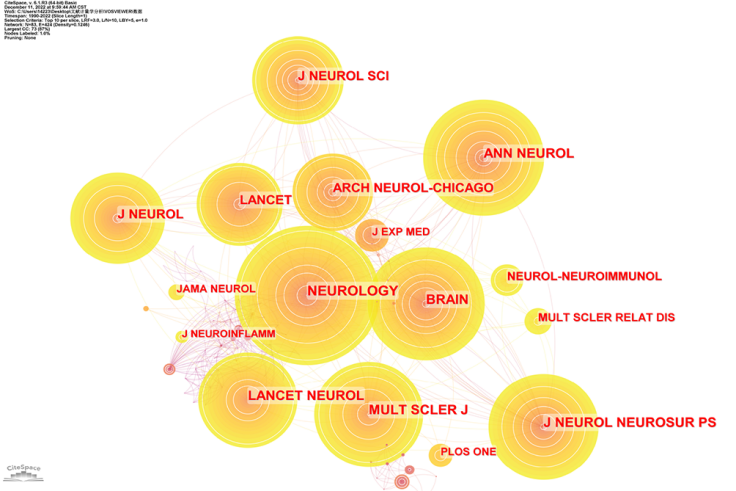


Supplementary Figure 5. Co-occurrence of co-cited journals involved in the study of NMOSD.


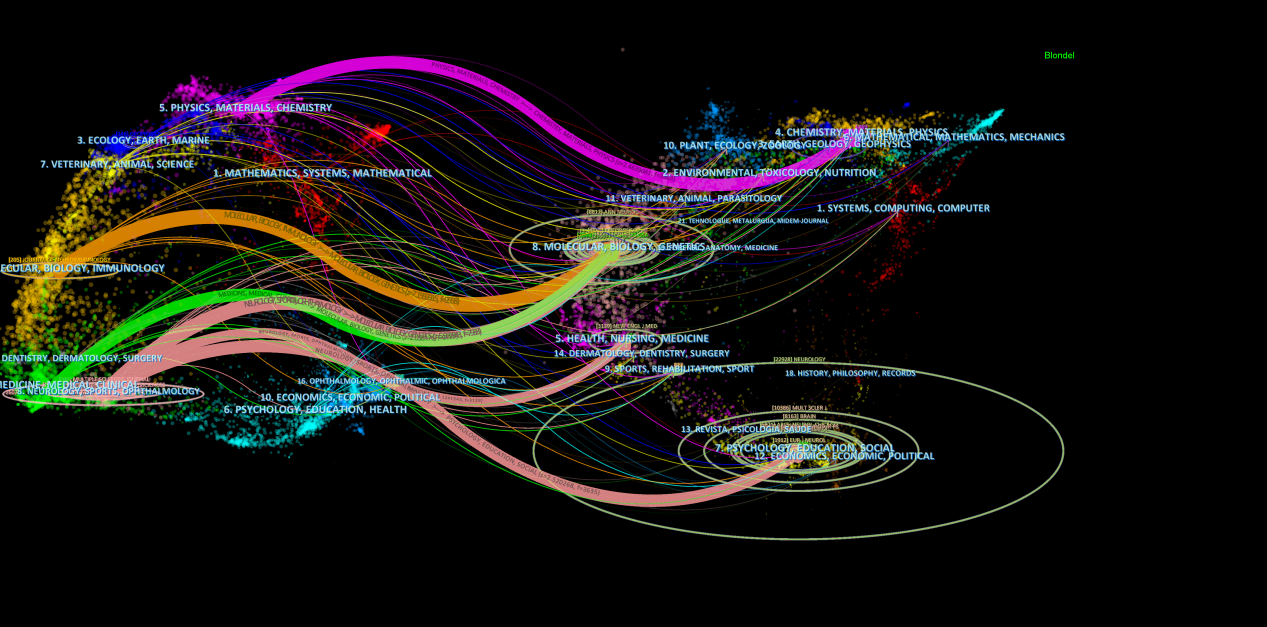


Supplementary Figure 6. A dual-map overlay of journals on NMOSD.


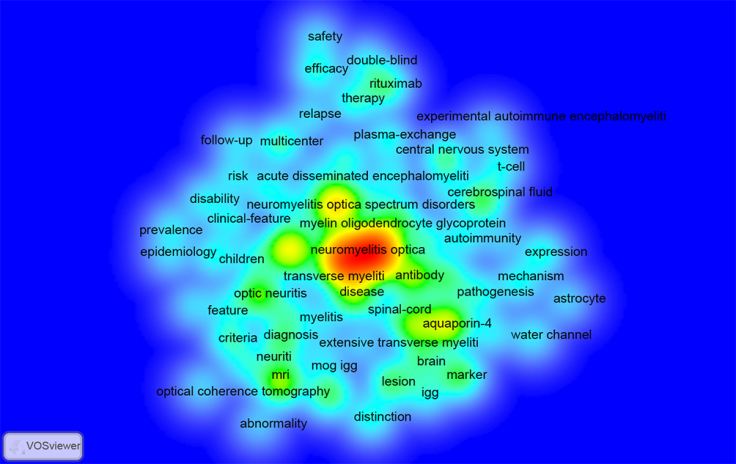


Supplementary Figure 7. Density visualization map of keywords appearing more than 40 times in the study of NMOSD.


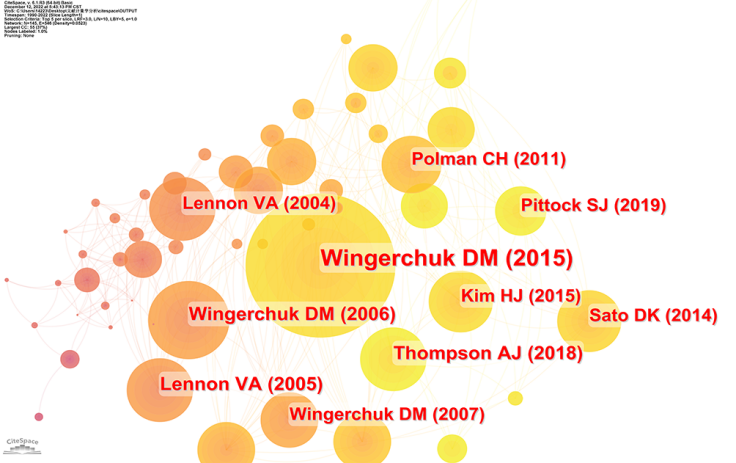


Supplementary Figure 8. Co-occurrence of co-cited references involved in the study of NMOSD.

Supplement Table 1. Top 10 co-cited authors of publications about NMOSD.

| Rank | Co-cited author | Country | Citations | TLS |
| --- | --- | --- | --- | --- |
| 1 | Wingerchuk, Dean M. | USA | 6333 | 31008 |
| 2 | Jarius, Sven | Germany | 3737 | 25786 |
| 3 | Lennon, Vanda A. | USA | 2808 | 18389 |
| 4 | Pittock, Sean J. | USA | 1923 | 15568 |
| 5 | Weinshenker, Brian G. | USA | 1037 | 8112 |
| 6 | Lucchinetti, Claudia F. | USA | 1030 | 7901 |
| 7 | Kitley, Joanna | England | 983 | 8241 |
| 8 | Kim, Sang-Hyun | South Korea | 969 | 7472 |
| 9 | Polman, Chris H. | Netherlands | 965 | 4269 |
| 10 | Misu, Tatsuro | Japan | 890 | 8169 |

Supplement Table 2. Top 10 co-cited journals of publications about NMOSD.

| Rank | Journal | Citations | IF（2021） | JCR |
| --- | --- | --- | --- | --- |
| --1 | NEUROLOGY | 22933 | 11.8 | Q1 |
| 2 | MULTIPLE SCLEROSIS JOURNAL | 10386 | 5.855 | Q1 |
| 3 | ANNALS OF NEUROLOGY | 8817 | 11.274 | Q1 |
| 4 | BRAIN | 8168 | 15.255 | Q1 |
| 5 | ARCHIVES OF NEUROLOGY | 5821 | 7.419 | Q1 |
| 6 | JOURNAL OF NEUROLOGY NEUROSURGERY AND PSYCHIATRY | 5517 | 13.654 | Q1 |
| 7 | JOURNAL OF NEUROLOGY | 4964 | 6.682 | Q1 |
| 8 | LANCET NEUROLOGY | 4955 | 59.935 | Q1 |
| 9 | JOURNAL OF THE NEUROLOGICAL SCIENCES | 4115 | 4.553 | Q2 |
| 10 | NEUROLOGY-NEUROIMMUNOLOGY & NEUROINFLAMMATION | 3977 | 11.36 | Q1 |

Supplement Table 3. Top 10 co-cited references of publications about NMOSD.

| Rank | Author | Title | Year | Journal | Citations |
| --- | --- | --- | --- | --- | --- |
| 1 | Wingerchuk, Dean M. | International consensus diagnostic criteria for neuromyelitis optica spectrum disorders | 2015 | NEUROLOGY | 1717 |
| 2 | Lennon, Vanda A. | A serum autoantibody marker of neuromyelitis optica: distinction from multiple sclerosis | 2004 | LANCET | 1662 |
| 3 | Wingerchuk, Dean M. | Revised diagnostic criteria for neuromyelitis optica | 2006 | NEUROLOGY | 1506 |
| 4 | Wingerchuk, Dean M. | The spectrum of neuromyelitis optica | 2007 | LANCET NEUROLOGY | 1173 |
| 5 | Lennon, Vanda A. | IgG marker of optic-spinal multiple sclerosis binds to the aquaporin-4 water channel | 2005 | JOURNAL OF EXPERIMENTAL MEDICINE | 1096 |
| 6 | Wingerchuk, Dean M. | The clinical course of neuromyelitis optica (Devic's syndrome) | 1999 | NEUROLOGY | 1002 |
| 7 | Lucchinetti, Claudia F. | A role for humoral mechanisms in the pathogenesis of Devic's neuromyelitis optica | 2002 | BRAIN | 647 |
| 8 | Polman, Chris H. | Diagnostic criteria for multiple sclerosis: 2010 revisions to the McDonald criteria. | 2011 | ANNALS OF NEUROLOGY | 571 |
| 9 | Kurtzke, John F. | Rating neurologic impairment in multiple sclerosis: an expanded disability status scale (EDSS). | 1983 | NEUROLOGY | 463 |
| 10 | Roemer, Shanu F. | Pattern-specific loss of aquaporin-4 immunoreactivity distinguishes neuromyelitis optica from multiple sclerosis | 2007 | BRAIN | 423 |
